# Foodborne Infections and Intoxications

**DOI:** 10.3201/eid1912.131335

**Published:** 2013-12

**Authors:** Henry Redel

**Affiliations:** Robert Wood Johnson University Hospital, New Brunswick, New Jersey, USA

**Keywords:** foodborne infections, foodborne intoxications, infectious diseases

The global supply of food has led to an increasingly connected planet, not only in terms of food products but also in terms of risks for foodborne diseases. The fourth edition of Morris and Potter’s Foodborne Infections and Intoxications delivers an in-depth look at the global effects of foodborne illnesses, provides pathogen-specific information, and describes processes and policies intended to prevent these illnesses. The text is a well-written and well-referenced guide to foodborne illnesses, containing contributions from >70 experts in epidemiology and the basic sciences of foodborne diseases.

The text has been organized into 6 sections. The first section describes the epidemiology of foodborne disease, highlighting the most common illnesses in the United States and abroad with country-specific data as well as the most common outbreak-associated foods and microbial risk assessment. The next 4 sections encompass microbe-specific illnesses; each section comprises chapters on specific pathogens. The sections are organized to include bacterial pathogens, viral pathogens, parasites (along with mycobacteria and prions), and agents involved with food intoxications. Each chapter is specific to a given pathogen and includes a clinical description of the disease, the microbiology of the pathogen, exposure risks, and disease prevention. 

Chapters have been written by leading researchers in their respective fields and are filled with up-to-date references on each pathogen. One example, in Chapter 8, includes a description of the 2011 outbreak of *Escherichia coli* O104:H4 infections in northern Germany. This outbreak illustrates the importance of food safety and the critical role of public health officials, clinicians, and scientists in identifying new outbreaks and limiting the spread of newly identified illnesses. The final section of the book outlines prevention of foodborne diseases and discusses food safety and the legal basis for food safety regulation.

This edition of Foodborne Infections and Intoxications updates the third edition, published in 2006, with increased emphasis on global disease prevention and a risk-based approach to food safety. This text is particularly valuable for students and practitioners in the fields of public health and food safety. It can also serve as a useful reference for public health investigators and officials.

